# Surgical Case Logging—Orthopaedic Application: A Narrative Review of Methods, Evolution, and Future Direction

**DOI:** 10.2106/JBJS.OA.25.00284

**Published:** 2026-04-09

**Authors:** Ala Alshomali, Mark Ehioghae, Sean Bae, Jonathan P. Japa, Aaron Phung, Noorullah Maqsoodi, Kevin Yoon, Justin Hyde, Jamie Lee, Addisu Mesfin

**Affiliations:** 1 Georgetown University School of Medicine, Washington, District of Columbia; 2 MedStar, Washington Hospital Center, Washington, District of Columbia; 3 Department of Orthopedic Surgery, Boston Medical Center Chobanian & Avedisian School of Medicine, Boston, Massachusetts; 4 Medstar Orthopaedic Institute, Georgetown University School of Medicine, Washington, District of Columbia

## Abstract

» Surgical case logs remain vital yet imperfect, often emphasizing volume over competency, and allowing data entry and coding inconsistencies.

» Lack of standardization across programs limits comparability and weakens the role of logs as true surgical competency measures.

» Case volume alone may not reflect surgical skill or autonomy, even among trainees meeting ACGME minimums.

» Emerging AI, EHR integration, and intraoperative analytics promise more accurate, efficient, and educationally valuable logging.

» The future is competency-based, linking procedural data with autonomy, complexity, and outcomes to better gauge readiness for independent practice.

## Introduction

Surgical case logging is a valuable proxy for gauging residents’ procedural experience and competency, fulfilling high-stakes certification requirements while maintaining patient safety and physician accountability. Accurate logs also help residency programs comply with duty-hour regulations introduced in 2003, which remain challenging for many surgical programs^1^. Unfortunately, current logging systems are prone to inaccuracy, lack standardization, and are vulnerable to fraudulent entries. Despite their importance, a survey of 18 orthopaedic residency programs found that only 53% of residents received formal case-log training^2^. This gap persists postresidency, as only 39% of orthopaedic fellows reported ever receiving formal case-log training^3^.

Traditional surgical logs often fail to capture case complexity, autonomy, or true learning progression. Duty-hour restrictions have decreased operative time for trainees, underscoring the need to evaluate surgical experience by competency rather than time^3^. This shift emphasizes the need for logging systems with greater granularity. Notably, no unified narrative or systematic review has examined the gaps in current surgical log systems. In this review, we examine existing surgical case-logging methods—particularly the Accreditation Council for Graduate Medical Education (ACGME's) case log—and assess their evolution, methodologies, limitations, and potential improvements. While emphasizing orthopaedic practices, we incorporate evidence from other surgical specialties to draw comparisons and highlight transferable innovations. The goal of this narrative review was to inform future modifications to orthopaedic surgical logging systems to improve standardization, accuracy, and technological proficiency, particularly in light of recent advancements in artificial intelligence (AI).

## Methods

We reviewed PubMed, OVID, and JBJS for articles published between 2010 and 2025 using the terms “Surgical Case Logging,” “Case Logging,” and “ACGME.” To maintain scope of this narrative review, exclusion criteria included nonsurgical specialties (e.g., anesthesiology or radiology), non-English language papers, commentaries, and opinion articles.

## Results

Of 1,106 records screened, 1,030 were excluded, 76 were sought for retrieval and 38 studies were included in the final review. Included studies were primarily focused on orthopaedic surgery (n = 17, 44.7%) and general surgery (n = 6, 15.8%). Cross-specialty or health-informatics–focused studies accounted for 18.4% (n = 7). The remaining studies represented pediatric surgery (n = 2), cardiothoracic surgery (n = 2), spine-focused studies (n = 2), vascular surgery (n = 1), and urology (n = 1). An observed trend in the literature is a greater concentration of studies within orthopaedic and general surgery compared with other surgical subspecialties; however, this distribution may reflect publication patterns rather than a comprehensive assessment of specialty-specific engagement.

## Historical Evolution

Before standardized residency accreditation, competency was assessed mostly through informal evaluations and subjective faculty feedback. Surgical cases were documented on paper or via program-specific mechanisms, creating substantial variability between programs and complicating assessments of resident readiness.^4^

**Fig. 1:**
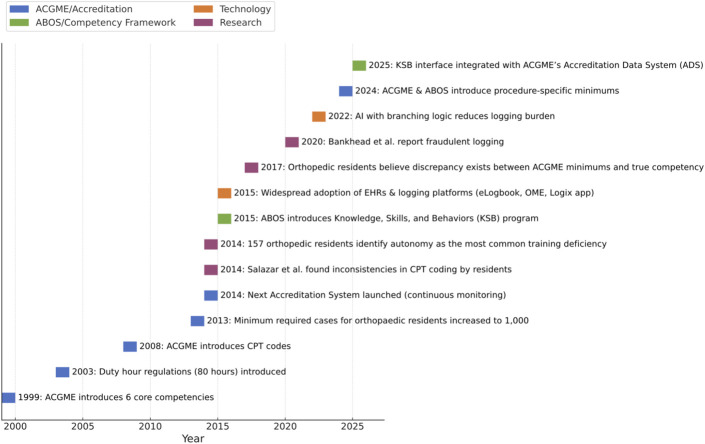
Chronological timeline of major events related to ACGME/accreditation, technological breakthroughs, ABOS/competency framework, and relevant research publications. ABOS =American Board of Orthopaedic Surgery, and ACGME = Accreditation Council for Graduate Medical Education.

In 1999, the ACGME introduced 6 core competencies to standardize residency evaluation: medical knowledge, patient care, professionalism, interpersonal and communication skills, practice-based learning and improvement, and systems-based practice (Fig. 1)^5^. This marked a shift from process-based to outcomes-based training. Despite improvements in evaluation, by 2003, growing concern over resident fatigue and patient safety led the ACGME to enforce an 80-hour work week limit^6^. This restriction reduced operative exposure, emphasizing the need for accurate case logging to track surgical experience under the new hours cap^6^.

In 2008, the ACGME introduced Current Procedural Terminology (CPT) codes into the logging system, establishing a standardized coding approach^3^. By 2015, widespread electronic health record (EHR) adoption coincided with the emergence of digitized platforms and adoption of AI (Fig. 1)^7,8^.

## Types of Case Logging Methods

### ACGME Case Log

The ACGME Resident Case Log is the primary system for documenting a resident’s surgical experience in the United States. Residents self-report each procedure by selecting the appropriate CPT code^9,10^. In 2015, the American Board of Orthopaedic Surgery (ABOS) introduced the Knowledge, Skill, and Behaviors program for competency-based assessment of resident’s case logs, with integration into ACGME Accreditation Data System planned for July 2025^10^.

### United Kingdom's eLogbook

The UK’s eLogbook is a self-reported electronic surgical log designed to provide accurate and secure documentation of surgical experience^11^. Similar to the ACGME system, a trainee’s eLogbook is reviewed annually by the Annual Review of Competency Progression committee to verify that yearly case requirements for advancement have been met^11^.

### Custom Databases (REDCap Platforms)

REDCap (Research Electronic Data Capture) is a secure web-based platform that can be adapted to create custom surgical logging databases^12,13^. For example, the Outcomes Management Evaluation database was designed for prospective documentation of shoulder arthroplasty procedures^12^. It emphasizes granular data input such as implant details and specific classifications (e.g., glenoid wear patterns, rotator cuff tear size, and bone graft use)^12^.

### Logix

Logix is a mobile app developed by Seadler et al. that exemplifies the shift toward competency-based evaluation in surgical training^7^. Unique to Logix, the interface allows trainees to log-specific steps of each surgery, creating a longitudinal record that emphasizes skill acquisition and autonomy^7^.

## Limitations and Challenges

### Data Entry Inconsistencies

Data entry errors in self-reporting surgical logs can include inaccuracies by unintentional omission or commission, causing logged information to deviate from the actual procedure performed^14^. Self-reported surgical logs (such as the ACGME system) require residents to designate themselves as either “primary surgeon” or “first assistant,” but the accuracy of these labels is questionable. In a survey of 363 general surgery residents, Bankhead-Kendall et al. found 94% could not correctly define “primary surgeon,” raising concerns about the consistency of self-categorization^15^.

Okike et al. found that orthopaedic residents at one institution failed to log 24% of cases, missed applicable CPT codes 46% of the time, and added extraneous CPT codes 28% of the time compared with the attending surgeons’ logs^16^.

Wide variability in CPT code selection further highlights data entry issues. In a 2014 study, Salazar et al. found broad inconsistencies among orthopaedic residents in CPT codes chosen for common procedures^17^. Similarly, McClure et al. presented a complex hypothetical spine surgery scenario to pediatric orthopaedic fellows, who selected between 2 and 15 CPT codes for the case (mean 6.34), underscoring a lack of standardization even among subspecialty-trained surgeons^3^. Another study found almost no correlation (r = –0.015) between residents' and attendings' CPT coding for foot and ankle procedures, indicating inconsistencies across experience levels^18^. Despite these issues, orthopaedic surgeons overall remain among the most accurate specialists in surgical logging^19^.

### Fraudulent Logging

Fraudulent logging involves intentional misrepresentation of surgical experience. Bankhead et al. identified this behavior among general surgery residents with 26% admitting they routinely selected a higher role—most often “primary surgeon”—when unsure if their involvement merited “first assistant”^14^. Residents cited motivations including boosting individual case counts (72%), augmenting low-volume categories (47%), improving program-wide statistics (37%), and compensating for limited primary surgeon opportunities due to faculty restrictions (37%)^14^.

In orthopaedic training, similar issues appear. McClure et al. reported 91% of orthopaedic fellows consistently logged cases where they were first assistants (as they should), but 9% did so inconsistently^3^. More concerning, 76% of fellows logged cases where they acted only as second assistants—a practice that could be considered fraudulent or at least misrepresentative in the context of surgical case logs^3^.

Such fraudulent logging behaviors can be detrimental to training. In cardiothoracic surgery, residents who admitted to logging cases they felt were unwarranted had lower self-reported in-training examination percentiles and felt less prepared for independent practice and board examinations^20^.

### Burden on Trainees and Faculty

The clerical burden of case logging and associated EHR documentation can detract from education and patient care^21,22^. In one survey, 90% of trainees reported that excessive electronic documentation time forced them to compromise direct patient care^21^. General surgery residents spend up to 24 hours per week on the EHR—nearly one-third of an 80-hour work week^21^. This workload contributes to burnout. A study of 1,781 faculty at one institution found that spending more than 90 minutes daily on the EHR outside of work hours was significantly associated with higher burnout (OR ∼1.65)^23^. Logging and documentation demands on faculty also limit their capacity to teach and evaluate. In orthopaedics, ACGME milestones require attendings to evaluate residents on 41 distinct topics (16 related to direct patient care), adding a considerable administrative load^24^.

### Validation and Impact

Collectively, the limitations above call into question the validity of using case logs as a proxy for operative competence. Volume-outcome relationships are well documented at the attending surgeon level—higher volumes are linked to better outcomes in many subspecialties^25,26^. For example, Schoenfeld et al. showed that for spinal metastasis surgeries, low-volume hospitals and surgeons had higher complication and readmission rates^25^. In the training context, however, this same volume-outcome relationship may not necessarily hold true. After the ACGME’s 2013 update, the minimum required cases for orthopaedic residents increased to 1,000, resulting in a 17% rise in mean logged procedures from 2014 to 2019 (1,464-1,709 cases on average), without reducing wide variability in operative experience among residents^27^.

In 2024, the ACGME and ABOS introduced new procedure-specific minimums emphasizing breadth across anatomical regions (Spine, 50) and depth in “3C” procedures (Common, Core, Competent), with defined case targets for core orthopaedic operations (Carpal tunnel releases, 20)^28^. Despite these refined requirements, concerns persist that operative experience may be diluted, as high-complexity procedures are increasingly replaced by lower-complexity cases that still satisfy many subspeciality minimums^29^. For example, both a complex 3-column lumbar osteotomy (CPT 22207) and a simpler single-column osteotomy (CPT 22214) count equally toward the 50-case spine requirement^28^. Such a scenario could further widen the gap between residency experience and early independent practice^30^.

Another key consideration is surgical autonomy. In a survey of 157 orthopaedic residents, the most commonly cited training deficiency was lack of autonomy in and out of the operating room^31^. In general surgery, 100% of residents achieved full autonomy after ∼25 laparoscopic appendectomies, but only 73.9% did so after 52 laparoscopic cholecystectomies—illustrating that case volume alone does not uniformly produce autonomy or proficiency^32^. Moreover, multiple studies report that residents believe they need more cases to achieve competence than the ACGME minimums mandate. For example, respondents reported needing approximately 53 spine decompression/posterior fusion cases (versus 15-case minimum), 51 total hip arthroplasties (vs. 30 required), and 37 ACL reconstructions (vs. 10 required) to feel competent^33^. Therefore, completing a predetermined number of procedures may not reliably indicate surgical competence^32^.

Researchers have also examined how case logs correlate with other competence measures, including board examinations. In neurosurgery, McGahan et al. found that the number of spine cases logged correlated with residents’ written board scores, but no such correlation was observed for other subspecialties^34^. Notably, neurosurgery residents perform on average 433.8 spine cases during training, compared with 119.5 for orthopaedic residents^35^. Further research is needed to identify whether orthopaedic subspecialties share a similar relationship.

**Fig. 2:**
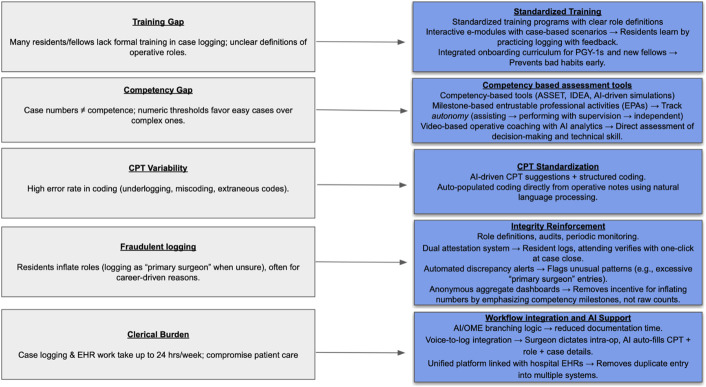
Key gaps in training, competency assessment, coding accuracy, integrity, and workload can be linked with corresponding innovations—including standardized education, competency-based tools, CPT standardization, audit systems, and AI-driven workflow integration—to improve the accuracy and educational value of surgical case logs. AI = artificial intelligence, and CPT = current procedural terminology.

Effective competence building in surgical training extends beyond procedural volume and requires alignment between documentation of operative experience and assessment of ability. Inadequate training in case logging and inconsistent operative role definitions contribute to variability in reported experience and obscure progression toward autonomy^2,3,15^. As portrayed in Fig. 2, standardized instruction in case logging, clear role definitions, and structured onboarding for early trainees are essential. When combined with competency-based assessment frameworks, such as milestone-linked entrustable professional activities and objective performance analytics, these measures can potentially support longitudinal evaluation of skill, judgment, and independence, better aligning competence acquisition with readiness for independent orthopaedic practice.

## Proposed and Emerging Improvements

### AI and automated capture

Manual surgical logging is time-consuming and prone to human error, but new platforms are leveraging AI to streamline and standardize the process. Thanawala et al. described an AI-enhanced logging system that automatically imports surgical case information from operating room schedules and synchronizes with updates made to the primary EHR postoperatively^8^. The system provides intelligent CPT code suggestions through AI-driven branching logic while preserving the user’s control to finalize entries.

Integrating this decision-support tool drastically reduced the time burden of logging and increased log completion. Residents logged an average of 4.77 ± 2.45 cases per week with the AI system, compared with 1.36 ± 1.3 with manual logging^8^. “Accuracy”—measured as the percentage of AI-suggested entries that residents accepted—reached 96% after 2,000 cases logged, indicating growing trust in the AI suggestions (though this metric reflects agreement with AI, not necessarily objective correctness)^8^.

## Simulation Tools and PROMs

In orthopaedics, several simulation-based tools are being validated. The Arthroscopic Surgery Skill Evaluation Tool differentiates skill levels in a diagnostic knee arthroscopy simulation, functioning as a pass/fail competency assessment^36^. Image-based decision error analysis evaluates wire navigation accuracy on fluoroscopic images during procedures such as hip fracture fixation^37^. More recently, AI-driven spinal surgery simulation has distinguished users by experience level using metrics including instrument angles, forces applied, and volume of virtual tissue resected^38^. These advances suggest future case logs could incorporate objective performance metrics, such as intraoperative force and motion data, to better track surgical skill progression (Fig. 2).

Connecting surgical logs to patient outcomes is essential for validating educational value and improving patient safety, yet current EHRs often overreport major complications and underreport minor ones^39^. To address this, Fisher et al. developed a web-based real-time complication tracking tool in pediatric surgery that increased reported complications 3.5-fold compared with the standard EHR (14 events vs. 4)^39^. Integrating Patient-Reported Outcome Measures (PROMs) with case logs may further connect surgical experience with patient-centered outcomes such as pain, function, or quality of life^40^.

## Discussion

Although surgical case logs are central to residency accreditation, training, and quality improvement, concerns about accuracy, validity, and educational impact remain. As surgical education shifts toward competency-based models, it is increasingly important to question whether current logging truly reflects operative competence or merely procedural exposure. Our review indicates a clear gap between the number of cases logged and actual surgical competence. Variability in CPT coding practices, subjective interpretation of operative roles, and inconsistent reporting undermine log reliability. Without deliberate integration of operative autonomy, case complexity, and patient outcomes, the current illusion of objectivity in case logs will likely persist.

These shortcomings have significant implications. Inflated or inaccurate case logs can misrepresent a graduate’s readiness for independent practice. Reliance on case log volume as a key metric may inadvertently promote a culture that values volume over the actual skills and expertise gained. In addition, the clerical burden on trainees and faculty diverts time from patient care and teaching, potentially diminishing care quality and contributing to burnout. However, advances in AI, especially in automated data extraction from the EHR with logic-based CPT and procedure suggestions, can markedly improve logging completeness and accuracy (Fig. 2).

While surgical case logs are often treated as procedural inventories, applying contemporary orthopaedic case-logging frameworks has the potential to evolve the whole practice of orthopaedic surgery. By emphasizing case complexity, autonomy, and PROMs (Fig. 2), logging systems can better reflect how orthopaedic surgeons treat their patients. In this context, case logs evolve from administrative records into practice-level representations of clinical performance and judgment. Such an approach aligns surgical education with real-world orthopaedic practice and has the potential to improve patient outcomes while producing residents who are better prepared for independent practice.

## Conclusion

Surgical case logs remain essential but flawed in surgical training. As education shifts toward competency-based models, reliance on volume-driven logging systems—often plagued by data entry errors, inconsistency, variability, and lack of granularity—is becoming increasingly misaligned with both accreditation standards and clinical needs. Low-technology solutions, such as structured and formalized training in accurate case logging practices, represent an immediately implementable strategy to enhance data quality, consistency, and educational value across training programs.
